# Psycho-Socio-Cultural Determinants of Delayed Presentation for Specialized Burn Care and Their Clinical Consequences: A Mixed Observational Study

**DOI:** 10.3390/jcm15062415

**Published:** 2026-03-21

**Authors:** Bogdan Oprita, Georgeta Burlacu, Vlad-Mircea Ispas, Cristina Virag-Iorga, Alice-Elena Diaconu, Ruxandra Oprita

**Affiliations:** 1 Emergency Department, Clinical Emergency Hospital of Bucharest, 050463 Bucharest, Romania; bogdan.oprita@umfcd.ro (B.O.); vlad.ispas.mail@gmail.com (V.-M.I.); 2 Faculty of Medicine, University of Medicine and Pharmacy “Carol Davila”, 050474 Bucharest, Romania; ruxandra.oprita@umfcd.ro; 3 Faculty of General Nursing, Bioterra University, 013724 Bucharest, Romania; 4 Clinical Emergency Hospital of Bucharest, 050463 Bucharest, Romania; iorga.cristina@yahoo.com; 5 Gastroenterology Department, Clinical Emergency Hospital of Bucharest, 050463 Bucharest, Romania

**Keywords:** burn injuries, first aid, health-seeking behavior, time of presentation, psychological impact, burn-induced pain, clinical outcomes

## Abstract

**Background**: Burn injuries have both physical and psychological impacts on patients. Factors such as personal beliefs, prior experiences, and geographic, economic, or cultural barriers, as well as fear of hospitals, can contribute to delays in seeking specialized care. When combined with inadequate first aid or the inappropriate use of pharmaceutical or traditional remedies, these delays may worsen burn severity, prolong healing, and negatively affect quality of life. From a clinical perspective, delayed presentation following burn injury has been linked to burn wound progression, which increases the risk of local infection, hypertrophic scarring and prolonged functional impairment. **Methods**: This analytical cross-sectional study was conducted at the Clinical Emergency Hospital of Bucharest between January and September 2025. The primary objective was to characterize adult burn patients presenting more than 24 h after injury (Group A) and to describe self-reported psychosocial/behavioral characteristics and explore unadjusted patterns among delayed presenters. Data were collected from medical records and a structured questionnaire administered to delayed presenters. A secondary descriptive comparison was performed with patients presenting within 24 h (Group B) to provide contextual reference. **Results**: The majority of patients were male (62.2%) and of working age (18–65 years, 82.4%). Thermal burns from domestic accidents were most common (58.8%), with scalds predominating. Second-degree burns were the most frequent (60.5%), primarily affecting the upper and lower limbs. Mean total body surface area (TBSA) was low (2.86 ± 1.91%), although higher values were observed in radiation burns and closed-space accidents. More than half of the patients did not receive any first aid, while the remainder used various pharmaceutical or natural products, some of which were inappropriate for burn treatment. The main reasons for delaying specialized care were the expectation that injuries would heal spontaneously, negligence, and fear of the hospital. In contrast, escalating pain, edema, and family insistence were the primary motivators for seeking professional medical attention. Delayed presentation was associated with older burn lesions, higher burn severity and an increased likelihood of hospitalization or refusal of recommended admission. **Conclusions**: Burn injuries predominantly affect working-age males and most frequently arise from domestic thermal accidents. Delayed presentation and inadequate first aid are common and influenced by behavioral, social, and demographic factors. Targeted public education, improved first aid practices, and timely healthcare-seeking are essential to reduce burn severity and improve patient outcomes.

## 1. Introduction

Burn injuries affect individuals across all age groups and both sexes, representing a substantial global health burden with widespread demographic distribution [1,2]. The consequences of burn trauma extend beyond physiological tissue damage to encompass significant psychological sequelae that influence emotional and cognitive functioning. The dynamic interplay between physical injury and psychological factors is integral to the recovery process, as these elements may either promote tissue repair and functional recovery or contribute to delayed healing and prolonged convalescence [3].

From a clinical standpoint, delayed presentation after burn injury represents a critical determinant of patient outcome. Even burns involving a limited total body surface area may undergo burn wound conversion when appropriate first aid and early medical evaluation are lacking. Delays beyond the first 24–48 h have been associated with progression of burn depth, increased risk of local infection, delayed epithelialization, hypertrophic scarring, prolonged functional impairment, and, in selected cases, the need for surgical intervention.

Immediately after the injury and throughout the therapeutic process, burn patients—and often their relatives—may experience a range of psychological disturbances, including acute stress reactions, anxiety, depression, and, in the longer term, post-traumatic stress disorder. In patients with visible scars, these psychological sequelae may be further compounded by reduced self-esteem, body image dissatisfaction, and social or interpersonal difficulties [4,5,6].

Partial-thickness burns that are initially perceived as minor can evolve into deeper injuries in the absence of adequate cooling, wound care, and early reassessment. Furthermore, delayed presentation often coincides with inappropriate first aid measures or the use of non-indicated pharmaceutical or traditional remedies, which may exacerbate tissue damage or mask early signs of deterioration.

Despite significant advances in burn management over recent decades, appropriate first aid and the immediate, prompt, and correctly applied initiation of specialized treatment remain of critical importance for optimal patient outcomes [7,8].

Depending on the stage of recovery and the severity of the burn injury, patients undergo different phases of psychological adaptation. This adaptation represents a complex and continuous process that often requires specialized, phase-specific interventions tailored to the individual patient [9]. It encompasses a range of psychological and cognitive–behavioral changes influenced by personal and contextual factors, including alterations in self-perception, anxiety, depressive symptoms, frustration, and difficulties in processing the traumatic experience [10].

The therapeutic management of burn patients is inherently multidisciplinary and complex, involving prompt emergency care, appropriate diagnostic evaluation, and a combination of autonomous and delegated medical interventions. In parallel, psychological support plays a crucial role throughout the recovery process. Therapeutic strategies should be integrated and adapted to the patient’s stage of recovery to facilitate psychological adjustment and prevent the development of chronic psychological sequelae. Interventions such as cognitive–behavioral therapy (CBT), grounded in cognitive and behavioral psychology principles, have demonstrated effectiveness in reducing anxiety, depression, and post-traumatic stress symptoms in burn patients [11].

From a healthcare system perspective, delayed burn presentation complicates clinical management, increases resource utilization, and may contribute to avoidable adverse clinical evolution. Understanding the psychosocial, cultural, and behavioral factors that influence patients’ decisions to postpone medical evaluation is therefore essential not only for prevention strategies but also for optimizing early clinical intervention and improving burn-related outcomes.

The primary aim of this study was to characterize the demographic, behavioral, psychosocial, and clinical features of adult burn patients presenting more than 24 h after injury.

A secondary aim was to perform an exploratory descriptive comparison between delayed presenters and patients presenting within 24 h, in order to contextualize observed patterns of injury characteristics and healthcare-seeking behavior.

### Research Gap

Although previous studies have examined clinical factors associated with delayed burn admission and focused primarily on burn severity, TBSA, and acute outcomes, limited research has systematically explored the psychosocial, behavioral, and cultural determinants influencing patients’ decisions to postpone medical evaluation. Furthermore, few prospective studies have simultaneously integrated demographic, behavioral, and clinical outcome variables within the same analytical framework. Therefore, the present study aims to address this gap by investigating non-medical observed patterns of delayed presentation and describing relationships observed between delay, burn characteristics, and short-term management indicators.

## 2. Materials and Methods

Study Design: This study was designed as an analytical cross-sectional investigation focusing primarily on adult burn patients presenting more than 24 h after injury. Data collection included both retrospective extraction of clinical information from medical records and prospective administration of a structured questionnaire to delayed presenters after ethical approval.

In addition, a secondary descriptive comparative component was included, involving patients presenting within 24 h of injury, to provide contextual reference for demographic and clinical characteristics. The study was reported in accordance with the STROBE (Strengthening the Reporting of Observational Studies in Epidemiology) guidelines for observational studies [12].

Efforts to minimize bias included the use of standardized medical records for clinical data extraction and structured, predefined questionnaire items administered uniformly to all eligible participants. Comparative analyses between groups were conducted using consistent variable definitions and identical measurement criteria.

Patient Selection: This study was conducted at the Clinical Emergency Hospital of Bucharest between January and September 2025. Of 366 burn patients presenting to the emergency department or transferred from other medical units, 119 (32.5%) met the inclusion criteria (Group A):

(a) Presence of burn lesions;
(b) Presentation to CEHB or another medical unit more than 24 h after the accident.

Delayed presentation was defined as hospital evaluation occurring more than 24 h after burn injury. This threshold was selected based on the previous literature indicating that early assessment within the first 24 h is critical to prevent burn wound progression and infectious complications [7,8,13]. Delays beyond this interval have been associated with increased burn depth conversion and suboptimal clinical outcomes.

A second group (Group B) comprised 180 patients who presented to the CEHB within <24 h of injury, sustained burn injuries of comparable severity to those observed in Group A and did not require hospital admission.

The third group (Group C) included patients admitted to the emergency department with major burns who required intubation and/or analgosedation and subsequent inpatient management. Owing to the severity of their injuries and overall clinical condition and incomplete comparable demographic data, these patients were excluded from analytical and comparative analyses.

Data Collection: Complete data were collected using structured questionnaires applied only for Group A (119 patients) and medical records, including:

(a) Demographics: age, gender, place of residence, and insurance status;
(b) Burn characteristics: etiology, type of accident, TBSA, depth, and lesion location;
(c) Healthcare-seeking behavior: time to presentation, first aid measures applied, pre-hospital assistance requested, reasons for delayed presentation, and reasons for hospital presentation;
(d) Hospitalization data: admission or refused admission, TBSA, burn severity, age of lesions, and clinical outcome.

For patients evaluated earlier during the study period, clinical data were extracted retrospectively from medical records. The structured questionnaire was administered only after ethical approval had been obtained (by telephone).

For patients presenting later during the study period, data collection was performed prospectively, and the questionnaire was administered either in person or by telephone.

The structured questionnaire was developed by a multidisciplinary team consisting of emergency physicians and one clinical psychologist, based on a targeted review of the literature addressing healthcare-seeking behavior and psychosocial determinants in burn patients, and ethical approval for its use was obtained from the institutional Ethics Committee prior to study initiation.

The instrument primarily consisted of single-item questions designed to capture specific contextual and behavioral factors (e.g., fear of hospital, perceived minor severity, work obligations). It included structured questions addressing: (1) the time elapsed since the burn injury; (2) the reasons for delayed hospital presentation; (3) first aid measures applied prior to medical evaluation; and (4) whether pre-hospital emergency assistance had been requested.

Prior to study initiation, the questionnaire was reviewed by the research team to ensure content relevance and clarity and was pilot-tested in a small number of patients to confirm comprehensibility and feasibility.

The questionnaire was administered by physicians to eligible patients presenting between January and September 2025.

The full list of questionnaire items is provided in the Supplementary Material. Responses were coded using predefined categorical options for descriptive analysis. Open-ended responses were grouped into thematic categories for reporting purposes.

As the instrument was not designed as a multi-item psychometric scale but rather as a structured clinical data collection tool, internal consistency reliability measures such as Cronbach’s α were not applicable.

Statistical analysis: Statistical analyses were primarily descriptive. Quantitative variables (age, TBSA, and time to presentation) were analyzed as continuous variables presented as the mean ± standard deviation or median (range), as appropriate, and categorical variables as counts and percentages. Comparisons between Group A and Group B were performed using appropriate parametric or non-parametric tests, depending on data distribution. Categorical variables were compared using appropriate contingency analyses. For generating the comparative table, continuous variables were analyzed using Welch’s Student *t*-test, and categorical variables were compared using the Chi-square test or Fisher’s exact test when expected cell frequencies were <5. Statistical significance was defined as *p* < 0.05. All analyses were considered exploratory.

Considering the exploratory objectives of the study and the limited number of hospitalization events, multivariate regression analysis was not performed. The statistical approach was therefore limited to descriptive and univariate analyses.

The statistical approach was selected in accordance with the exploratory objectives of the study. Descriptive statistics were used to characterize the study population, while correlation analyses and group comparisons were performed to explore potential associations between delay and clinical variables.

Statistical analyses were performed using IBM SPSS Statistics, version SPSS 25.0 (IBM Corp., Armonk, NY, USA).

Variables: For analytical purposes, time to presentation was analyzed descriptively as a grouping variable (≤24 h vs. >24 h). No predictive modeling was performed and analyses were limited to descriptive and exploratory comparisons between groups.

Outcomes: The primary outcome of the study was the characterization of demographic, behavioral, and clinical features of patients presenting more than 24 h after burn injury.

Secondary outcomes included the association between time to presentation and indicators of burn severity (TBSA, burn depth), as well as the requirement for hospitalization or refusal of recommended admission.

Sample Size Considerations: No formal sample size calculation was performed prior to study initiation. The study included all consecutive adult patients presenting more than 24 h after burn injury during the predefined study period (January–September 2025). The sample size was therefore determined by the number of eligible cases within this timeframe. The analysis was considered exploratory in nature.

## 3. Results

### 3.1. Baseline Characteristics and Comparison Between Group A and Group B

A total of 299 patients were included in the comparative analysis: 119 in Group A (>24 h) and 180 in Group B (≤24 h). Baseline demographic and clinical characteristics are summarized in Table 1.

All eligible patients presenting during the study period were included in the analysis. No patients were excluded due to incomplete essential clinical data. Complete data were available for all analyzed clinical variables in both groups.

All comparative analyses were exploratory in nature.

Patients in Group A and Group B demonstrated comparable mean ages (46.24 ± 14.29 vs. 44.54 ± 13.47 years; *p* = 0.304). A significantly higher proportion of males was observed among delayed presenters (62.18% vs. 48.33%; *p* = 0.019). Rural residence was more frequent in Group A (37.82% vs. 26.11%; *p* = 0.032).

Burn extent, expressed as mean TBSA, was similar between groups (2.86 ± 1.91% vs. 2.95 ± 1.94%; *p* = 0.692).

Hospitalization was recommended more frequently in Group A compared with Group B (12.61% vs. 4.44%; *p* = 0.010). Refusal rates did not differ significantly between groups (*p* = 0.392), while hospital admission acceptance occurred exclusively among delayed presenters (*p* = 0.001).

#### 3.1.1. Demographics

**Figure 1:**
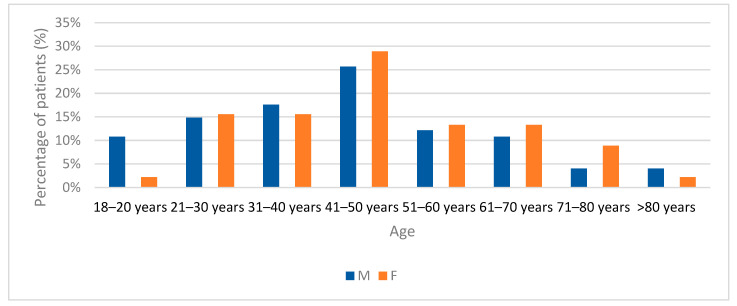
Distribution of patients by age groups.

The study population for Group A was predominantly male (62.2%, *n* = 74), with a male-to-female ratio of 1.64:1. The mean age of patients was 46.24 ± 14.29 years (range of 18–99 years), with female patients having a slightly higher mean age (48.13 ± 14.42 years; range of 19–99 years) than male patients (45.08 ± 14.32 years; range of 18–93 years). The age group with the highest representation was 41–50 years, both in the overall cohort and within gender-specific subgroups (Figure 1). Most patients (82.4%, *n* = 98) were of working age (18–65 years). The majority of patients resided in urban areas (62.2%, *n* = 74), and most were medically insured (73.9%, *n* = 88).

**Figure 2:**
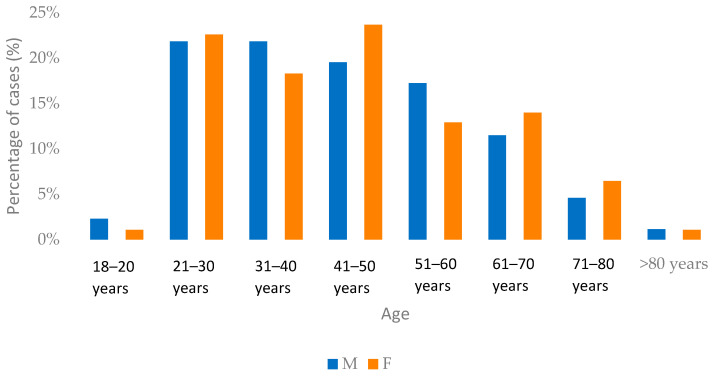
Distribution of patients from Group B by age groups.

In comparison, the male-to-female ratio in Group B was 0.94:1, with a relatively higher proportion of female patients (51.67%, *n* = 93). The mean age was 44.54 ± 13.47 years (range: 18–90 years), with the highest proportional representation observed in the 21–50-year age groups. Most patients resided in urban areas (73.89%, *n* = 133). Health insurance coverage was documented in 88.33% of cases (*n* = 159). (Figure 2).

#### 3.1.2. Etiology and Context

**Figure 3:**
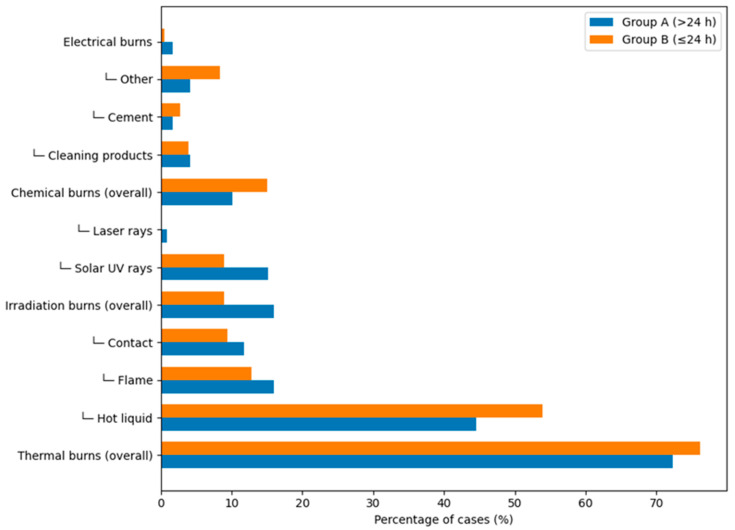
Detailed comparative distribution of burn etiology in Group A (>24 h) and Group B (≤24 h).

**Figure 4:**
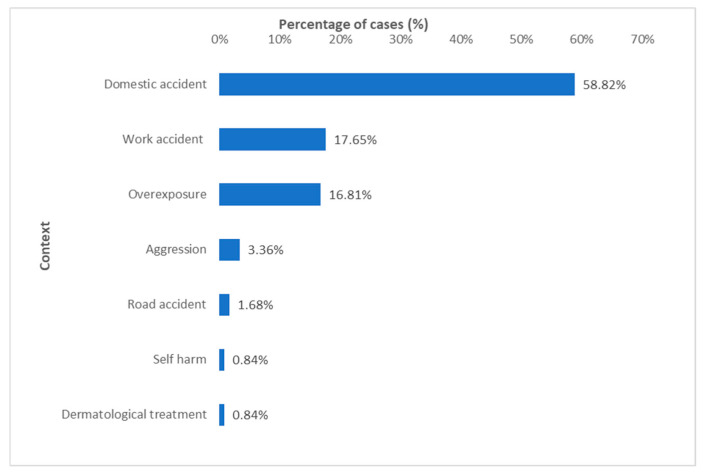
Context of burn injuries.

Thermal burns were the most common type of injury, accounting for 72.27% of cases (*n* = 86) in Group A and 75% in Group B, with the majority resulting from hot liquids, including water, oil, and tea (Figure 3). Most burns occurred in the context of domestic accidents. However, work-related accidents and prolonged exposure to radiation sources (e.g., UV rays, lasers, and heat sources) also contributed significantly (Figure 4), but with a smaller proportion in the second group (16.67% and 8.33%). The vast majority of accidents (90.9%) occurred in open spaces, with only one patient sustaining burns from an explosion (gas stove cylinder) in an open environment in Group A.

#### 3.1.3. Severity of Burn Injuries

In Group A, the mean total body surface area (TBSA) affected was 2.86 ± 1.91% (range of 0.5–15%). The majority of patients (82.4%, *n* = 98) presented with limited burns involving ≤4% TBSA, while injuries exceeding 10% TBSA were uncommon (1.7%, *n* = 2). Slight differences in mean TBSA were observed according to sex and residence, with marginally higher values among male and rural patients; however, overall burn extent remained low in most cases. Detailed stratified analyses are provided in Supplementary Table S1 and Supplementary Figure S2.

When analyzed by injury mechanism, radiation-related burns—particularly those associated with prolonged UV exposure—demonstrated the highest mean TBSA values within this group. Although burns acquired in open environments occasionally reached higher TBSA values, overall differences between open and closed settings were minimal. Further details according to injury context are presented in Supplementary Table S2.

Regarding burn depth, second-degree burns predominated in Group A (60.5%, *n* = 72), followed by third-degree burns (28.6%, *n* = 34) and first-degree burns (10.9%, *n* = 13). Higher proportions of deeper burns were observed among patients with chemical and electrical injuries, as well as in selected injury contexts (Supplementary Tables S3–S6).

In Group B, the mean TBSA was comparable (2.95 ± 1.94%, range of 0.5–18%), with burns ≥10% TBSA identified in 4.44% of cases (*n* = 8). Second-degree burns were the predominant injury type (89.44%, *n* = 161), whereas third-degree burns were infrequent (1.67%, *n* = 3). No clear association between age and TBSA was observed within this cohort.

#### 3.1.4. Location of Burn Injuries

**Figure 5:**
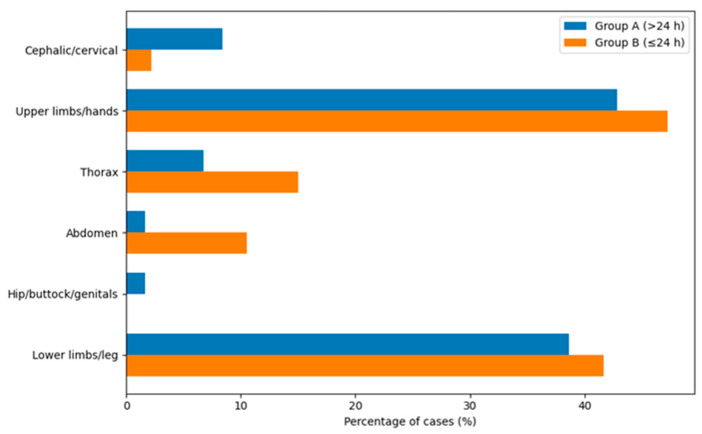
Comparative distribution of burn location in Group A and Group B.

In both groups, burn injuries most commonly involved the upper limbs (42.9% and 47.22% respectively) and lower limbs (38.7% and 41.67% respectively) (Figure 5). A sex-related difference was observed in burn location, with female patients exhibiting a higher proportion of cephalic and/or cervical burns compared to male patients for Group A analysis (see Supplementary Figure S1).

#### 3.1.5. Time to Presentation

Group A

The mean interval between burn injury and presentation to a medical facility was 5.01 ± 3.49 days. Differences in time to presentation were observed across demographic subgroups, with longer delays noted among male patients, individuals residing in rural areas, and particularly among uninsured patients (Table 2).

Patients with chemical burns exhibited the longest delays in presentation to a medical facility, particularly those resulting from improper use of topical medical ointments (e.g., Fastum Gel) or household cleaning agents, such as chlorine, sodium-based products, and descaling agents. When analyzed according to the context of injury occurrence, the longest mean intervals between burn injury and hospital presentation were observed among patients injured in road traffic accidents and those sustaining burns in domestic settings (Table 3).

The longest mean intervals between burn occurrence and presentation to a medical facility were observed in patients with burns affecting the upper and lower limbs (6.80 ± 5.22 days and 4.90 ± 3.30 days, respectively; range of 1–30 days). In contrast, burns located in other anatomical regions were associated with considerably shorter times to presentation (3.25 ± 1.41 days; range of 1–7 days).

Group B

The mean presentation time for this patient group was 3.52 ± 2.54 h (range: 1–14 h). No major differences were observed between the mean presentation times across patient subgroups. However, relatively longer intervals were noted among patients residing in rural areas and among those older than 60 years (see Supplementary Table S7).

Patients sustaining flame burns demonstrated the shortest mean interval between injury occurrence and hospital presentation. Conversely, patients with burns secondary to prolonged UV radiation exposure and those with chemical burns recorded the longest mean presentation intervals (Table 4).

The longest delays to presentation were observed in patients with burns involving the cephalic/cervical region (9.75 ± 2.38 h), thorax (6.33 ± 4.30 h), and abdomen (5.58 ± 4.04 h). In contrast, burns affecting the upper and lower extremities were associated with significantly shorter mean presentation times (2.72 ± 1.86 h and 3.12 ± 1.87 h, respectively).

All analyses within Group B are descriptive. Inferential comparisons are presented only in the direct comparison between Group A and Group B.

### 3.2. Detailed Findings Among Delayed Presenters (Group A)

#### 3.2.1. First Aid Measures

More than half of the patients (50.42%) did not apply any first aid measures following the burn injury. Non-application of first aid was more frequently observed among female patients (53.33%), individuals residing in urban areas (52.70%), and uninsured patients (64.52%). When stratified by age, the highest proportion of patients who did not receive first aid was noted in the 18–30-year age group (59.26%).

Among patients who did apply first aid, water was the most commonly used measure (23.53%). An exception was observed in patients younger than 30 years, who more frequently reported the use of pharmaceutical products marketed for burn treatment (e.g., Baneocin, Cicatridin spray, calendula cream, Oximed, propolis-based ointments), even though not all of these agents are indicated for the initial management of burn injuries.

The use of pharmaceutical products without an established indication for burn care (e.g., Advantan, Aerius, Betadine) was more prevalent among female patients, those residing in rural areas, uninsured individuals, and patients under 30 years of age. Natural remedies with purported indications for burns, as well as contraindicated substances such as yogurt or vinegar, were used less frequently (1.68% of all patients).

Burns for which first aid measures were least frequently applied were sunburns (38.89%), followed by burns associated with hot liquids (43.40%). Water was most used as a first aid measure in cases of burns associated with household cleaning products (80%) and flame-related injuries (36.84%). Although high proportions of first aid use were also observed in cases of cement burns (50%) and electrical injuries (50%), these findings should be interpreted with caution due to the small number of patients in each category (*n* = 2 per category).

When analyzed by type and context of the accident, burns resulting from prolonged exposure to radiation or heat sources and those sustained in domestic accidents were the most frequently neglected, with 65.0% and 51.4% of patients, respectively, reporting no first aid measures. Burns occurring in enclosed spaces were also commonly underestimated by patients. Interpretation for other accident types is limited due to small subgroup sizes. Water was the most frequently used first aid measure in work-related accidents (47.6%), whereas patients involved in other types of accidents more often reported using pharmaceutical products or traditional remedies.

No significant association was identified between burn extent and the application of first aid measures. Interpretation of findings in patients with burns involving more than 9% TBSA is limited due to the small number of cases in these categories, precluding meaningful statistical inference. With respect to burn depth, both first-degree and third-degree burns were frequently neglected, with a large proportion of patients reporting no application of first aid measures (61.50% of patients presenting this type of burn).

Burns involving the upper and lower limbs were more frequently neglected with respect to first aid application, a finding that parallels the longer delays observed in presentation for these injury locations. In contrast, patients were less likely to use water as a first aid measure for burns affecting the head and cervical region (Table 5).

#### 3.2.2. Pre-Hospital Assistance and Professional Recommendations

Among the 119 patients included in the study, 10.1% (*n* = 12) reported requesting pre-hospital emergency services (ambulance). Additionally, 10.9% (*n* = 13) consulted their family physician, while 37.0% (*n* = 44) sought guidance from a pharmacist prior to presentation at a medical facility.

#### 3.2.3. Causes of Delayed Hospital Presentation

The primary reason reported by patients for delaying hospital presentation was negligence, expressed through statements such as “I thought it would pass” (26.05%) or “I did not consider it important.” (18.49%). Additionally, 13.5% of patients cited fear of the hospital as a contributing factor. The remaining patients cited work or family obligations, medical or logistical constraints that limited access to hospital care, or a preference for consulting a family physician or pharmacist.

As shown in Table 6, male patients and those residing in urban areas were more likely to postpone hospital presentation due to negligence or the expectation that the burns would heal spontaneously. In contrast, fear of the hospital was more frequently reported by female patients, individuals from rural areas, and uninsured patients.

Analysis of average ages within each demographic subgroup revealed that older patients, those living in urban areas, and insured individuals often cited comorbid conditions (e.g., diabetes) or lack of transportation as reasons for delayed presentation. Patients who reported being constrained by work obligations were younger on average yet still presented to the emergency department within a maximum of 3 days post-injury (mean 2.3 days).

Analysis of time to presentation revealed that patients who initially sought advice from a pharmacist or their family physician experienced the longest delays before presenting to the hospital (13.75 ± 8.25 days, range of 1–30 days). Assuming that these consultations occurred promptly after the burn injury, this finding suggests either that the guidance provided was insufficient to prompt timely hospital presentation or that patients did not adhere fully to the treatment instructions received.

#### 3.2.4. Reasons for Presenting to the Hospital

**Figure 6:**
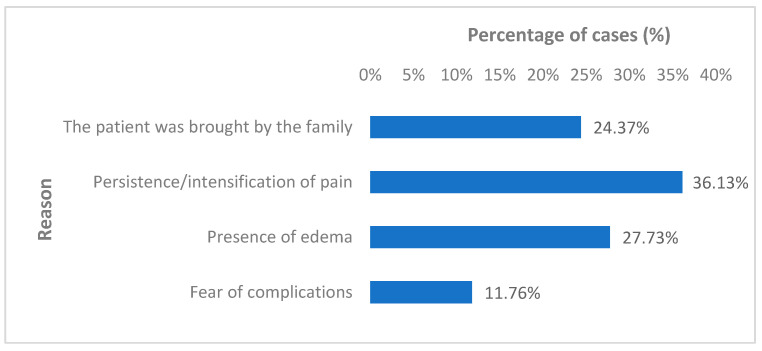
Reasons for presenting to the hospital.

The most frequently reported reason for seeking hospital care was persistent or increasing pain. Edema and family insistence were also commonly cited as motivating factors for presentation (Figure 6).

Analysis of patient demographics revealed that across all categories—particularly among uninsured individuals—a substantial proportion sought hospital care primarily due to pain. Age-related differences were minimal, except that patients with the highest mean age typically required family assistance to present to the emergency department.

Patients who delayed hospital presentation the longest, on average, were those who reported fear of potential complications as their motivating factor (mean time to presentation of 6.57 ± 5.31 days, range of 1–30 days). Similarly, individuals citing pain as the primary reason for seeking care also exhibited a prolonged mean interval between injury and hospital presentation (mean time to presentation of 5.09 ± 3.63 days, range of 1–30 days).

The majority of patients (87.4%, *n* = 104) presented with burn injuries and a general condition that did not require hospitalization, while 6.72% refused hospitalization. Hospitalization was both recommended and accepted for 5.88% of patients (*n* = 7). The mean age of hospitalized patients was 53.6 ± 20.2 years (range of 23–99 years), with burns averaging 4.71 ± 3.67 days old (range of 1–12 days) and a mean TBSA of 5.86% ± 2.94% (range of 1–15%). Most injuries were second- or third-degree burns. All hospitalized patients were victims of domestic accidents, with six of seven sustaining thermal burns from either flame or scalding.

Among hospitalized patients, reasons for delayed presentation included fear of the hospital (*n* = 3), negligence (*n* = 3), and inability to travel (*n* = 1). Five patients were brought to the hospital by family members; one presented due to pain, and another due to fear of complications.

In 6.7% of cases (*n* = 8), hospitalization was recommended but refused. The mean age in this group was 52.3 ± 8.4 years (range of 41–80 years), and the group was predominantly male (7/8), with five patients lacking health insurance. Burns in this group had a mean age of 3 ± 2.5 days (range of 1–7 days) and a mean TBSA of 4.94% ± 3.30% (range of 0.5–10%), with primarily second- and third-degree injuries. Most were thermal burns; one case each involved chemical injury and electrocution, occurring in domestic or occupational contexts. Delays in presentation were attributed to negligence, work obligations, or inability to travel, with only one patient citing fear of the hospital. Five patients were brought by family members, while three self-presented due to pain.

#### 3.2.5. Clinical Outcomes Associated with Delayed Presentation

Although most patients presented with burns of limited extent, delayed presentation was associated with clinically relevant outcomes. Hospitalization was recommended in 12.6% of cases, including patients who ultimately refused admission. Among hospitalized patients, burns were older, involved a larger mean TBSA and were more frequently second- or third-degree injuries compared to the overall cohort.

Patients requiring hospitalization had a mean burn age of 4.71 ± 3.67 days and a mean TBSA of 5.86 ± 2.94%, indicating that delayed presentation was associated with more advanced local injury at the time of medical evaluation. All hospitalized cases correlated with domestic accidents and delayed presentation was commonly attributed to negligence or fear of the hospital.

Furthermore, patients who refused recommended hospitalization frequently lacked health insurance and presented with clinically significant burns, suggesting that delayed care may contribute not only to injury progression and reduced acceptance of appropriate medical management.

## 4. Discussion

In this study, we characterized adult burn patients presenting more than 24 h after injury and explored differences compared with patients presenting within 24 h. The findings suggest that delayed presentation is associated with distinct demographic patterns, contextual factors, and differences in healthcare engagement.

Consistently with previous reports, the majority of burn patients were male and predominantly of working age [14,15]. Urban residence was more common overall, likely reflecting population distribution and referral patterns to tertiary centers. Although age distribution did not differ substantially between sexes, males represented a larger proportion of cases across most age categories. Similar sex-related patterns in burn epidemiology have been described in prior studies and may reflect differences in occupational exposure and activity profiles [7,16,17]. In our cohort, female patients were slightly older on average and more frequently presented with burns involving the head and cervical region, possibly reflecting differences in domestic exposure contexts.

With respect to timing of presentation, male patients in the delayed group tended to present slightly later than females, although the absolute difference was modest. The previous literature has suggested that work-related obligations, perceived time constraints, and competing priorities may influence healthcare-seeking behavior [18,19]. While our study was not designed to evaluate these mechanisms directly, the observed patterns are consistent with existing models of healthcare utilization.

Residence and insurance status differed between cohorts. Patients presenting within 24 h more frequently resided in urban areas and reported health insurance coverage. These observations may reflect differences in healthcare accessibility [20,21]. Consequently, poor socio-economic conditions can produce psychosocial barriers, reducing the individual’s capacity to recognize health needs and increasing delays in accessing care.

Thermal and scald injuries were the most common etiologies in both groups, occurring primarily in domestic and occupational settings. Limb involvement predominated, particularly affecting the upper extremities. Injuries affecting the extremities and burns of limited TBSA were common among delayed presenters. Psychosocial factors such as agitation, anxiety, fatigue, and psychological stress are associated with impaired cognitive functioning and accident proneness, which in turn increase the risk of unintentional injuries [22,23]. Such injury patterns may be perceived as less severe at the time of occurrence; however, this study did not directly assess patient perception.

Psychological and social considerations may contribute to variability in healthcare-seeking behavior. The prior literature has documented associations between burn injuries and psychological distress, including anxiety and depressive symptoms, which may influence coping strategies and decision-making processes [24,25]. Fear of medical evaluation and pain-related avoidance behaviors have also been described as potential contributors to delayed presentation [26,27]. Although psychological scales were not administered in the present study, family involvement was frequently reported as a trigger for eventual hospital attendance, suggesting that social context may influence care-seeking decisions.

Patients presenting after 24 h more frequently exhibited deeper burns at the time of evaluation, despite comparable overall TBSA between groups. While delayed assessment may coincide with more advanced local wound characteristics, the present study cannot establish temporal causality. These findings should therefore be interpreted within the descriptive and exploratory framework of the analysis.

First aid practices varied between groups. Immediate cooling with water was reported more frequently among early presenters, whereas delayed presenters more often described self-initiated treatment using non-indicated products or household remedies. These observations highlight differences in initial injury management, although the study design does not allow evaluation of their direct clinical impact.

From a conceptual perspective, healthcare-seeking behavior in burn injuries may be interpreted through health behavior frameworks, such as the Health Belief Model, which emphasize perceived severity, perceived barriers, and cues to action. In cases of limited-appearing burns, lower perceived severity combined with logistical or emotional barriers may contribute to delayed evaluation. This interpretation remains theoretical and was not formally tested in the present study.

Overall, the comparative findings between late and early presenters are descriptive and hypothesis-generating. They suggest observable differences in demographic characteristics, injury context, and patterns of healthcare engagement, which warrant further investigation in analytically designed studies.

### Strengths and Limitations

This study has several limitations that should be considered when interpreting the results. First, the research was conducted in a single tertiary emergency center, which may limit the generalizability of the findings to other healthcare settings or populations. Second, although two cohorts were analyzed, the comparison was exploratory and not designed as a controlled analytical study with multivariable adjustment. Also, the overall sample size was adequate for descriptive analysis, but the number of patients with larger burn extent, deeper burns, or specific injury mechanisms was relatively small. This limited the statistical power of certain subgroup analyses and correlation assessments, particularly those involving burn extent, burn depth, and time to presentation.

Additionally, some of the observed extreme correlation coefficients did not reach statistical significance and are likely influenced by small subgroup sizes or aggregated data, rather than representing true underlying associations. Therefore, these findings should be interpreted cautiously and viewed as exploratory.

The questionnaire was administered partly retrospectively and, in some cases, by telephone, which may have introduced recall bias. In addition, self-reported reasons for delayed presentation may be influenced by social desirability bias, as patients may underreport negligence or overemphasize socially acceptable explanations.

Despite these limitations, the study has several strengths, including its design, detailed characterization of burn injuries and healthcare-seeking behavior, and the integration of clinical, demographic, and psychosocial variables and offers comparative context with early presenters. These features provide valuable insights into delayed presentation for burn care and characterize patients presenting with delayed evaluation.

The statistical analyses were exploratory and should be interpreted cautiously, particularly in subgroup analyses with limited sample size.

## 5. Conclusions

In this study, nearly one-third of adult burn patients presented to the burn center more than 24 h after injury. Most delayed presentations involved burns of limited TBSA, predominantly second-degree injuries affecting the extremities. These findings indicate that delayed evaluation may occur even in cases perceived as clinically minor at the time of injury.

Comparative analysis with patients presenting within 24 h showed that, although overall burn extent was similar between groups, late presenters exhibited a higher proportion of deeper burns at the time of assessment. Differences were also observed in demographic patterns, residence distribution, and first aid practices, suggesting variation in healthcare engagement and timing of medical evaluation between cohorts [7].

Overall, the findings suggest that delayed presentation may relate to contextual factors beyond burn extent.

From a practical perspective, delayed presentation may represent an important modifier of burn presentation profiles and should be carefully considered during clinical assessment and triage. Further research is warranted to clarify underlying determinants and to develop targeted strategies aimed at improving timely access to specialized burn care.

The study provides a descriptive characterization of patients presenting after 24 h and highlights observable differences compared with early presenters. These findings are exploratory in nature and should be interpreted cautiously.

## Figures and Tables

**Table 1:** Comparison between patients presenting >24 h (Group A) and <24 h (Group B) after injury.

Variable	Group A (>24 h) (*n* = 119)	Group B (<24 h) (*n* = 180)	*p*-Value	Statistical Test
*Demographics*				
Age (years), mean ± SD	46.24 ± 14.29	44.54 ± 13.47	0.304	Welch Student *t*-test
Male sex, *n* (%)	74 (62.18)	87 (48.33)	0.019	Chi-square
Rural residence, *n* (%)	45 (37.82)	47 (26.11)	0.032	Chi-square
*Injury characteristics*				
TBSA %, mean ± SD	2.86 ± 1.91	2.95 ± 1.94	0.692	Welch Student *t*-test
*Management and outcome*				
Hospitalization recommended, *n* (%)	15 (12.61)	8 (4.44)	0.010	Chi-square
Hospitalization refusal, *n* (%)	8 (6.72)	8 (4.44)	0.392	Chi-square
Hospitalization accepted, *n* (%)	7 (5.88)	0	0.001	Fisher exact

**Table 2:** Time to presentation by patient categories.

Patient Group	Mean Time to Presentation	Range
Total study group	5.01 ± 3.49 days	1–30 days
Males	5.11 ± 3.54 days	1–30 days
Females	4.84 ± 3.46 days	1–30 days
Rural	5.40 ± 4.11 days	1–30 days
Urban	4.77 ± 3.15 days	1–30 days
With health insurance	4.44 ± 2.85 days	1–30 days
Without health insurance	6.55 ± 5.39 days	1–30 days

**Table 3:** Time to presentation according to etiology and context *.

Etiology	Mean Time to Presentation	Range	Context	Mean Time to Presentation	Range
Thermal burns	4.80 ± 3.08 days	1–30 days	Domestic accident	5.11 ± 3.32 days	1–30 days
*Hot liquid*	4.32 ± 2.64 days	1–14 days	Work accident	2.81 ± 1.78 days	1–7 days
*Flame*	6.11 ± 4.75 days	1–30 days	Overexposure	4.65 ± 2.94 days	1–30 days
*Contact*	4.86 ± 2.43 days	1–14 days	Aggression	5 ± 3 days	2–9 days
Irradiation burns	3.05 ± 1.01 days	1–6 days	Road accident	17 ± 13 days	4–30 days
*Solar UV rays*	3.11 ± 1.02 days	1–6 days	Self-harm *	30 days	100%
Laser rays *	2 days		Dermatological treatment *	2 days	
Chemical burns	9.75 ± 10.17 days	1–30 days			
*Cleaning products*	7.80 ± 8.88 days	1–30 days	Open space	4.98 ± 3.53 days	1–30 days
*Cement*	2.50 ± 0.50 days	2–3 days	Closed space	5.27 ± 3.02 days	1–12 days
*Other*	14.60 ± 12.32 days	1–30 days			
Electrical burns	4 ± 3 days	1–7 days			

* Percentages are calculated within category; small subgroups should be interpreted cautiously.

**Table 4:** Time to presentation according to etiology and context (Group B) *.

Etiology	Mean Time to Presentation (Hours)	Range (Hours)	Context	Mean Time to Presentation (Hours)	Range (Hours)
Thermal burns	2.55 ± 1.46	1–12	Domestic accident	3.02 ± 11.87	1–12
*Hot liquid*	2.64 ± 1.49	1–12	Work accident	2.10 ± 1.02	1–6
*Flame*	1.91 ± 0.95	1–6	Overexposure	10.73 ± 1.56	2–14
*Contact*	2.88 ± 1.63	1–10			
Irradiation burns (*UV rays*)	10.73 ± 1.56	2–14			
Chemical burns	4.41 ± 3.21	1–12			
*Cleaning products*	5.86 ± 4.69	1–12			
*Cement*	4.00 ± 2.40	1–10			
*Other*	3.87 ± 2.64	1–12			
Electrical burns *	3				

* Percentages are calculated within category; small subgroups should be interpreted cautiously.

**Table 5:** First aid measures vs. burn lesion location.

	Cephalic/ Cervical	Upper Limbs/Hands	Thorax	Abdomen *	Hip/Buttock/ Genitals	Lower Limbs/Legs
Water		20.00%	20.00%	100.00%		19.35%
Pharmaceutical products with indication for burns	28.57%	11.43%				9.68%
Pharmaceutical products without indication for burns	28.57%	5.71%	20.00%			3.23%
Natural products indicated for burns		2.86%				3.23%
Others (yogurt, vinegar)					100.00%	3.23%
Nothing	42.86%	60.00%	60.00%			61.29%

* One patient.

**Table 6:** Causes of delayed hospital presentation vs. demographics.

Causes of Delayed Hospital Presentation	Sex		Residence		Health Insurance	
	M	F	Urban	Rural	With Health Insurance	Without Health Insurance
“I thought it would pass.”	29.73%	20.00%	29.73%	20.00%	27.27%	22.58%
Negligence/”I did not consider it important.”	20.27%	15.56%	21.62%	13.33%	18.18%	19.35%
Fear of the hospital	10.81%	17.78%	10.81%	17.78%	10.23%	22.58%
“I was out of town.”	10.81%	13.33%	10.81%	13.33%	13.64%	6.45%
“I was at work.”	8.11%	8.89%	8.11%	8.89%	11.36%	45.16%
Family obligations	6.76%	11.11%	6.76%	11.11%	7.95%	9.68%
Conditions or circumstances that prevented the patient from traveling to the hospital	8.11%	6.67%	8.11%	6.67%	6.82%	9.68%
“I went to the pharmacy or family doctor.”	4.05%	2.22%	1.35%	6.67%	3.41%	3.23%
Refused medical assistance	1.35%	4.44%	2.70%	2.22%	1.14%	6.45%
Total	100%	100%	100%	100%	100%	100%

## Data Availability

The de-identified clinical and questionnaire data supporting the findings of this study are available from the corresponding author upon reasonable request, subject to institutional approval and compliance with ethical and data protection regulations.

## Supplementary Materials

The following supporting information can be downloaded at https://www.mdpi.com/article/10.3390/jcm15062415/s1.
