# Supplementary material for: Psycho-Socio-Cultural Determinants of Delayed Presentation for Specialized Burn Care and Their Clinical Consequences: A Mixed Observational Study
Source: J Clin Med. 2026 Mar 21;15(6):2415. doi: 10.3390/jcm15062415 (PMC13026473; doi:10.3390/jcm15062415)
Supplement: Supplementary file 1 [file jcm-15-02415-s001.zip › Supplementary Material S1.pdf]

Structured Questionnaire Used in the Study

This structured questionnaire was administered to adult burn patients presenting more than 24 hours after injury.

The instrument consisted of four clinically oriented anamnestic questions designed for clarity and ease of comprehension.

1. Time Elapsed Between Burn Injury and Hospital Presentation

Question:
How much time elapsed between the burn injury and your presentation to the hospital?

Response format: Numerical value recorded in hours or days.

2. Reasons for Delayed Presentation

Question:
What were the reasons for not presenting earlier to the hospital?

Response format (predefined categories; multiple responses allowed where applicable):

- Perceived minor severity of the burn
- Negligence / underestimation of symptoms
- Fear of hospital treatment or medical procedures
- Work-related or personal obligations
- Advice from family or acquaintances
- Other (specified by the patient)

Open-ended responses were categorized thematically for descriptive analysis.

3. First Aid Measures Applied Prior to Medical Evaluation

Question:
What first aid measures did you apply before coming to the hospital?

Response format (categorical):

- Application of cold water
- Application of topical substances (e.g., creams, home remedies)
- No intervention
- Other (specified by the patient)

4. Pre-Hospital Emergency Assistance

Question:
Did you request pre-hospital emergency assistance?

Response format:

- Yes
- No

<u>Response Coding</u>

Categorical responses were coded numerically for descriptive statistical analysis. Open-ended responses were grouped into thematic categories when appropriate. Time-to-presentation values were recorded as continuous variables.
