# Supplementary material for: Psycho-Socio-Cultural Determinants of Delayed Presentation for Specialized Burn Care and Their Clinical Consequences: A Mixed Observational Study
Source: J Clin Med. 2026 Mar 21;15(6):2415. doi: 10.3390/jcm15062415 (PMC13026473; doi:10.3390/jcm15062415)
Supplement: Supplementary file 1 [file jcm-15-02415-s001.zip › Supplementary Material Table S1.pdf]

Table S1. TBSA according to the etiology and context of burn injuries

Etiology	Mean TBSA%)	Range (%)	Context	Mean TBSA%)	Range (%)
**Thermal burns**	**2.66±1.75**	**0.5-15**	**Domestic accident**	2.51±1.52	0.5-15
Hot liquid	*2.72±1.55*	*0.5-10*	**Work accident**	2.93±2.33	0.5-10
Flame	*3.68±2.73*	*0.5-15*	**Overexposure**	4.58±2.13	0.5-10
Contact	*1.04±0.41*	*0.5-2*	**Aggression**	1.88±1.13	0.5-4
**Irradiation burns**	**4.71±2.09**	**0.5-10**	**Road accident**	1.50±0.50	1-2
Solar UV rays	*4.72±1.98*	*0.5-10*	**Self-harm***	0.5	0.5
*Laser rays**	*1*	*1*	**Dermatological treatment***	1	1
**Chemical burns**	**1.63±0.92**	**0.5-4**			
Cleaning products	*1.40±0.64*	*1-3*			
Cement	*2.50±1.50*	*1-4*			
Other	*1.50±0.80*	*0.5-3*			
**Electrical burns**	**1.50±0.50**	**1-2**			

* one patient
