# Supplementary material for: Psycho-Socio-Cultural Determinants of Delayed Presentation for Specialized Burn Care and Their Clinical Consequences: A Mixed Observational Study
Source: J Clin Med. 2026 Mar 21;15(6):2415. doi: 10.3390/jcm15062415 (PMC13026473; doi:10.3390/jcm15062415)
Supplement: Supplementary file 1 [file jcm-15-02415-s001.zip › Supplementary Material Table S2.pdf]

Table S2.1. Depth of burn injuries

Depth	M	F	Rural	Urban
**1st degree**	8.11%	15.56%	6.67%	13.51%
**2nd A-B degree**	63.51%	55.56%	66.67%	56.76%
**3rd degree**	28.38%	28.89%	26.67%	29.73%

S2.2. According to patients' age *

Age group	1st degree	2nd A-B degree	3rd degree
**18-20 years**	22.22%	66.67%	11.11%
**21-30 years**	16.67%	61.11%	22.22%
**31-40 years**	15.00%	50.00%	35.00%
**41-50 years**	12.50%	56.25%	31.25%
**51-60 years**	0.00%	80.00%	20.00%
**61-70 years**	7.14%	64.29%	28.57%
**71-80 years**		42.86%	57.14%
**>80 years**		75.00%	25.00%

* The calculation of the percentage weights was done by referring to the number of patients in every age group
