# Supplementary material for: Psycho-Socio-Cultural Determinants of Delayed Presentation for Specialized Burn Care and Their Clinical Consequences: A Mixed Observational Study
Source: J Clin Med. 2026 Mar 21;15(6):2415. doi: 10.3390/jcm15062415 (PMC13026473; doi:10.3390/jcm15062415)
Supplement: Supplementary file 1 [file jcm-15-02415-s001.zip › Supplementary Material Table S3.pdf]

Table S3 Burn depth according to etiology and context of injury*

Etiology	1st degree	2nd A-B degree	3rd degree	Context	1st degree	2nd A-B degree	3rd degree
**Thermal burns**	**1.16%**	**68.60%**	**30.23%**	**Domestic accident**	1.43%	64.29%	34.29%
Hot liquid	*1.89%*	*71.70%*	*26.42%*	**Work accident**		85.71%	14.29%
Flame		*68.42%*	*31.58%*	**Overexposure**	60.00%	25.00%	15.00%
Contact		*57.14%*	*42.86%*	**Aggression**		75.00%	25.00%
**Irradiation burns**	**63.16%**	**31.58%**	**5.26%**	**Road accident**			100%
Solar UV rays	*66.67%*	*27.78%*	*5.56%*	**Self-harm***			100%
*Laser rays**		*100%*		**Dermatological treatment***		100%	
**Chemical burns**		**50.00%**	**50.00%**				
Cleaning products		*80.00%*	*20.00%*	**Open Space**	12.04%	62.04%	25.93%
Cement			*100%*	**Closed space**		45.45%	54.55%
Other		*40.00%*	*60.00%*				
**Electrical burns**		**50.00%**	**50.00%**				

* The calculation of the percentage weights was done by referring to the number of patients in every group defined by burn etiology and context.
** one patient
