# Supplementary material for: Psycho-Socio-Cultural Determinants of Delayed Presentation for Specialized Burn Care and Their Clinical Consequences: A Mixed Observational Study
Source: J Clin Med. 2026 Mar 21;15(6):2415. doi: 10.3390/jcm15062415 (PMC13026473; doi:10.3390/jcm15062415)
Supplement: Supplementary file 1 [file jcm-15-02415-s001.zip › Supplementary Material Table S4.pdf]

Table S4. TBSA according to the etiology and context of burn injuries (Group B).

Etiology	Mean TBSA%)	Range (%)	Context	Mean TBSA%)	Range (%)
**Thermal burns**	**2.75±1.74**	**0.5-18**	**Domestic accident**	2.65±1.78	0.5-18
Hot liquid	*3.06±1.86*	*0.5-18*	**Work accident**	2.72±1.31	0.5-7
Flame	*2.54±1.45*	*0.5-7*	**Overexposure**	6.07±2.87	1-11
Contact	*1.26±0.70*	*0.5-4*			
**Irradiation burns** *(UV rays)*	**6.07±2.87**	**1-11**			
**Chemical burns**	**2.24±1.49**	**0.5-10**			
Cleaning products	*2.71±2.45*	*0,5-10*			
Cement	*2.10±0.72*	*0.5-3*			
Other	*2.07±1.30*	*0.5-6*			
**Electrical burns***	**2**				

* one patient.
