# Supplementary material for: Psycho-Socio-Cultural Determinants of Delayed Presentation for Specialized Burn Care and Their Clinical Consequences: A Mixed Observational Study
Source: J Clin Med. 2026 Mar 21;15(6):2415. doi: 10.3390/jcm15062415 (PMC13026473; doi:10.3390/jcm15062415)
Supplement: Supplementary file 1 [file jcm-15-02415-s001.zip › Supplementary Material Table S5.pdf]

Table S5. Depth of burn injuries (Group B).

S5.1. According to patients' sex and residency

Depth	M	F	Rural	Urban
**1st degree**	9.20%	8.60%	8.51%	9.02%
**2nd A-B degree**	88.51%	90.32%	89.36%	89.47%
**3rd degree**	2.30%	1.08%	2.13%	1.50%

S5.2. According to patients' age *

Age group	1st degree	2nd A-B degree	3rd degree
**18-20 years**		66.67%	33.33%
**21-30 years**	20.00%	80.00%	
**31-40 years**	5.56%	94.44%	
**41-50 years**	12.82%	84.62%	2.56%
**51-60 years**		100.00%	
**61-70 years**		100.00%	
**71-80 years**	10.00%	80.00%	10.00%
**>80 years**		100.00%	

* The calculation of the percentage weights was done by referring to the number of patients in every age group.
