# Supplementary material for: Psycho-Socio-Cultural Determinants of Delayed Presentation for Specialized Burn Care and Their Clinical Consequences: A Mixed Observational Study
Source: J Clin Med. 2026 Mar 21;15(6):2415. doi: 10.3390/jcm15062415 (PMC13026473; doi:10.3390/jcm15062415)
Supplement: Supplementary file 1 [file jcm-15-02415-s001.zip › Supplementary Material Table S6.pdf]

Table S6. Burn depth according to etiology and context of injury (Group B)*.

Etiology	1st degree	2nd A-B degree	3rd degree	Context	1st degree	2nd A-B degree	3rd degree
**Thermal burns**	**4.38%**	**94.16%**	**1.46%**	**Domestic accident**	6.67%	92.59%	0.74%
Hot liquid	5.15%	93.81%	1.03%	**Work accident**		93.33%	6.67%
Flame		100.00%		**Overexposure**	46.67%	53.33%	
Contact	5.88%	88.24%	5.88%				
**Irradiation burns** *(UV rays)*	**50.00%**	**50.00%**					
**Chemical burns**	**11.11%**	**85.19%**	**3.70%**				
Cleaning products	14.29%	85.71%					
Cement	20.00%	60.00%	20.00%				
Other	6.67%	93.33%					
**Electrical burns***		**100.00%**					

* The calculation of the percentage weights was done by referring to the number of patients in every group defined by burn etiology and context.
