# Supplementary material for: Psycho-Socio-Cultural Determinants of Delayed Presentation for Specialized Burn Care and Their Clinical Consequences: A Mixed Observational Study
Source: J Clin Med. 2026 Mar 21;15(6):2415. doi: 10.3390/jcm15062415 (PMC13026473; doi:10.3390/jcm15062415)
Supplement: Supplementary file 1 [file jcm-15-02415-s001.zip › Supplementary Material Table S7.pdf]

Table S7. Time to presentation by patient categories (Group B).

Patient group	Mean time to presentation (hours)	Range (hours)
**Total Group B**	3.51±2.54	1 – 14
**Males**	3.51±2.55	1 – 12
**Females**	3.52±2.54	1 – 14
**Rural**	4.09±2.72	1 – 12
**Urban**	3.31±2.44	1 – 14
**With health insurance**	3.58±2.22	1 – 14
**Without health insurance**	3.58±2.22	1 – 12
**18-20 years**	2.67±1.56	1 – 5
**21-30 years**	3.53±2.81	1 – 12
**31-40 years**	3.11±2.40	1 – 12
**41-50 years**	3.67±2.41	1 – 14
**51-60 years**	3.37±2.15	1 – 12
**61-70 years**	4.04±3.11	1 – 12
**71-80 years**	3.80±2.76	1 – 12
**>80 years**	3.00±1.00	2 – 4
