# Supplementary material for: Psycho-Socio-Cultural Determinants of Delayed Presentation for Specialized Burn Care and Their Clinical Consequences: A Mixed Observational Study
Source: J Clin Med. 2026 Mar 21;15(6):2415. doi: 10.3390/jcm15062415 (PMC13026473; doi:10.3390/jcm15062415)
Supplement: Supplementary file 1 [file jcm-15-02415-s001.zip › Supplementary Material Table S8.pdf]

Table S8. Professional recommendations*

	Pharmacy recommendations	Family doctor recommendations
**Advantan**	9.09%	
**Baneocin**	9.09%	
**Betadine**	13.64%	7.69%
**Cicatridine**	11.36%	15.38%
**Fenistil**	2.27%	
**Oximed**	29.55%	46.15%
**Raniseptol**	15.91%	7.69%
**Regenon**	31.82%	46.15%

* The calculation of the percentage weights was done by referring to the number of patients who consulted pharmacists or family doctors
