# Supplementary figures and images for: Psycho-Socio-Cultural Determinants of Delayed Presentation for Specialized Burn Care and Their Clinical Consequences: A Mixed Observational Study
Source: J Clin Med. 2026 Mar 21;15(6):2415. doi: 10.3390/jcm15062415 (PMC13026473; doi:10.3390/jcm15062415)

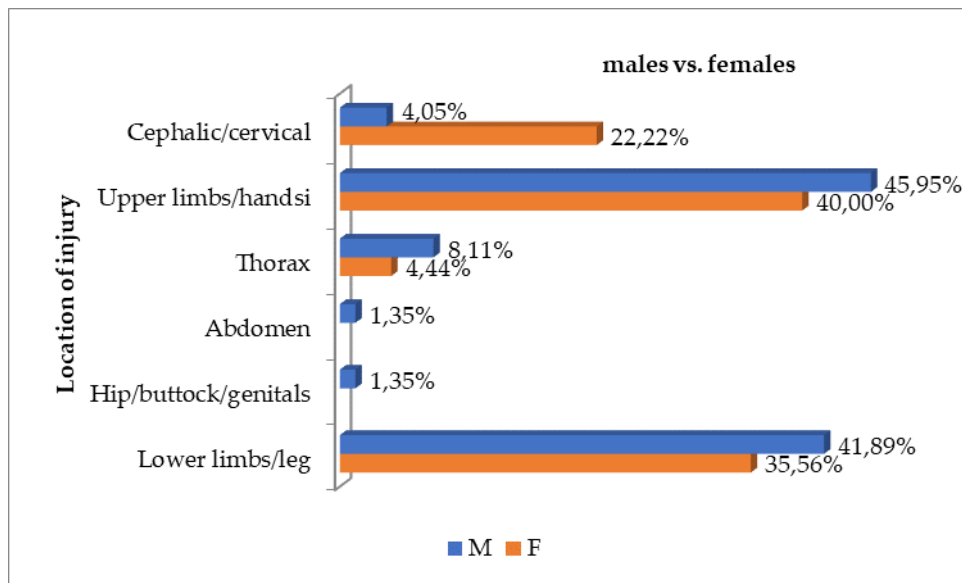

**Figure S1.** Location of burn injuries – male vs. female

Supplement: Supplementary file 1 [file jcm-15-02415-s001.zip › Supplementary Material Figure S1.pdf]

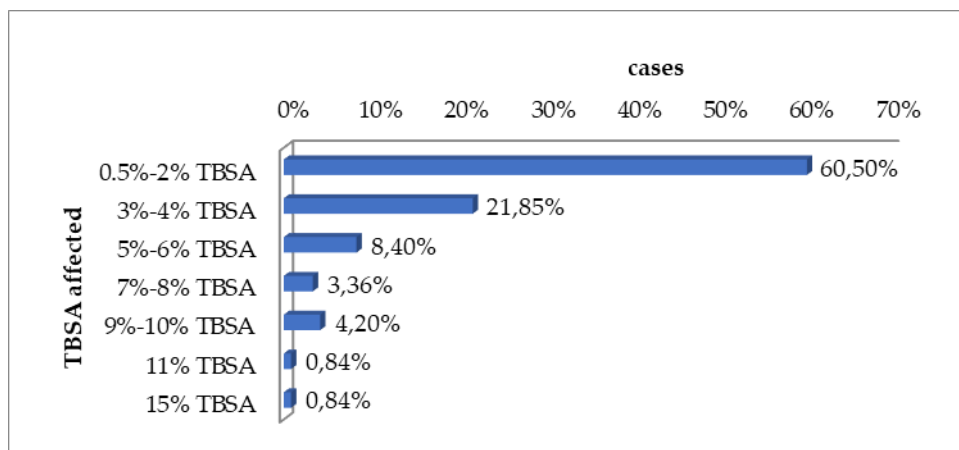

**Figure S2.** Extension of burn injuries

Supplement: Supplementary file 1 [file jcm-15-02415-s001.zip › Supplementary Material Figure S2.pdf]
